# Severe Asthma and Active SARS-CoV-2 Infection: Insights into Biologics

**DOI:** 10.3390/biomedicines13030674

**Published:** 2025-03-10

**Authors:** Sara Manti, Michela Leotta, Federica D’Amico, Simone Foti Randazzese, Giuseppe Fabio Parisi, Salvatore Leonardi

**Affiliations:** 1 Pediatric Unit, Department of Human Pathology in Adult and Developmental Age “Gaetano Barresi”, University of Messina, Via Consolare Valeria, 1, 98124 Messina, Italy; sara.manti@unime.it (S.M.); michela.leotta@studenti.unime.it (M.L.); federica.damico1@studenti.unime.it (F.D.); 2 Pediatric Respiratory Unit, Department of Clinical and Experimental Medicine, University of Catania, Via Santa Sofia, 78, 95123 Catania, Italy; gf.parisi@policlinico.unict.it (G.F.P.); leonardi@unict.it (S.L.)

**Keywords:** asthma, adults, children, biologics, antiviral effects, safety, SARS-CoV-2

## Abstract

Since the onset of the COVID-19 pandemic, managing asthma has become significantly more challenging. Both national and international guidelines emphasize the importance of continuing prescribed medications to maintain asthma control and prevent exacerbations. However, the emergence of SARS-CoV-2 infection has raised concerns about the safety of biologic therapies during acute COVID-19 episodes, necessitating a careful and individualized approach to their use. Biologic therapies, including omalizumab, dupilumab, mepolizumab, reslizumab, benralizumab, and tezepelumab, which target specific pathways in severe asthma, have revolutionized asthma management by improving symptom control and reducing exacerbation rates. Despite their proven benefits, the intersection of biologic therapy and active SARS-CoV-2 infection has prompted questions regarding potential immunomodulatory effects and risks. This review aimed to synthesize the current literature on the antiviral effects and safety of biologic drugs in severe asthmatic patients with active SARS-CoV-2 infection, encompassing both pediatric and adult populations.

## 1. Introduction

Since the onset of the coronavirus disease 2019 (COVID-19) pandemic, managing chronic conditions such as asthma has become increasingly challenging. Asthma treatment guidelines recommend therapies tailored to disease severity, including inhaled corticosteroids (ICSs), long-acting bronchodilators, anti-leukotrienes, and biologic therapies for severe asthma [1].

Maintaining optimal asthma control and preventing exacerbations were critical goals during the pandemic. Both national and international guidelines emphasized the importance of adhering to prescribed treatments to sustain disease control. For patients with severe asthma, continuing biologic therapy was strongly encouraged, with home administration recommended as a practical option to reduce hospital visits and minimize exposure to SARS-CoV-2. In cases of acute COVID-19 infection, clinicians were advised to carefully assess whether discontinuing biologic therapy was necessary [1,2].

However, discontinuing biologic treatment may lead to poor asthma control, increasing the risk of severe exacerbations. Accordingly, several studies proposed continuing treatment with biologics in patients with severe acute respiratory syndrome coronavirus 2 (SARS-CoV-2) infection [3,4].

Nevertheless, to date, open questions about the antiviral effects and safety of biologic drugs in treating patients with chronic diseases, such as asthma, and documented SARS-CoV-2 infection have arisen. There is a need to balance the risks and benefits of continuing these therapies amidst an acute viral illness.

This review aimed to provide a comprehensive overview of the use of biologic drugs in asthmatic patients affected by SARS-CoV-2 infection.

Relevant articles were identified through a systematic search conducted using the PubMed database, Global Health, EMBASE, Web of Science, Google Scholar, BMJ Best Practice, National Institute for Health and Care Excellence, and the World Health Organization (WHO). The following variations and terms were used: “biologic drugs”, “biological”, “monoclonal antibody”, “omalizumab”, “mepolizumab”, dupilumab”, “reslizumab”, “benralizumab”, “tezepelumab”, “COVID-19”, “SARS-CoV-2”, “asthma”, “exacerbation”, “safety”, “adverse events”, “child”, “children”, “adolescent”, and “adult”.

Evidence-based guidelines from the major scientific societies, reviews, meta-analyses, systematic reviews, original articles, case series, case reports, and letters to the editor published between January 2014 and December 2024 were included to capture the latest evidence and clinical insights.

## 2. Severe Asthma in the Context of COVID-19: Challenges and Uncertainties

Emerging in December 2019 in Wuhan, China, COVID-19 rapidly spread to nearly every country worldwide, leading to a dramatic rise in hospitalizations due to pneumonia, acute respiratory distress syndrome (ARDS), multisystem organ involvement, and death [5].

The variability in the clinical outcomes of COVID-19, ranging from asymptomatic cases to severe conditions, is not yet fully understood. Endemic human coronaviruses share significant genetic similarities with SARS-CoV-2, which may contribute to cross-reactive immune responses and potentially explain milder cases of COVID-19 in some individuals [6].

SARS-CoV-2 enters the host by binding to angiotensin-converting enzyme 2 (ACE2) receptors and using transmembrane protease serine 2 (TMPRSS2), initially infecting nasal epithelial cells and later spreading to the lower respiratory tract. This triggers an inflammatory immune response, involving both innate and adaptive mechanisms, including humoral and cell-mediated immunity [7].

Individuals with asthma might be expected to have higher susceptibility to SARS-CoV-2 infection and severe COVID-19 due to impaired antiviral immune responses and their predisposition to exacerbations triggered by respiratory viruses. Initially, the classification of asthmatic patients and those with other chronic lung conditions as high-risk groups for COVID-19 was based on precaution rather than robust scientific evidence. However, current data have not consistently demonstrated a higher prevalence of SARS-CoV-2 infection among asthmatic subjects [8].

Severe asthma, affecting 5–10% of the approximately 300 million people with asthma worldwide, is associated with increased mortality, frequent hospitalizations, reduced quality of life (QoL), and elevated healthcare costs. According to the International European Respiratory Society (ERS)/American Thoracic Society (ATS) guidelines, severe asthma is defined as “asthma which requires treatment with high dose inhaled corticosteroids (ICS) plus a second controller (and/or systemic corticosteroids) to prevent it from becoming ‘uncontrolled’ or which remains ‘uncontrolled’ despite this therapy”. This challenging condition often requires complex management strategies that include additional therapies, such as biologics, to achieve adequate control [9,10].

Based on the major immune–inflammatory pathway involved in severe asthma, type 2 (T2)-high, T2-low, and mixed endotypes are described [11,12].

Driven by T helper 2 (Th2) cells and T2 cytokines, such as interleukin 4 (IL-4), IL-5, and IL-13, the T2-high asthma endotype involves eosinophils, which may provide partial protection against COVID-19 by suppressing T1 inflammatory responses and cytokine storms. ICS reduces ACE2 expression and may lower SARS-CoV-2 susceptibility. Despite a reduced eosinophil presence in severe COVID-19, eosinophil recovery correlates with clinical improvement [13,14].

The T2-low asthma endotype is characterized by neutrophilic inflammation and T helper 17 (Th17)/IL-17 pathways and is associated with severe airway remodeling and resistance to anti-inflammatory treatments. Higher ACE2 expression in these patients may increase COVID-19 susceptibility. Neutrophils and neutrophil extracellular traps (NETs) contribute to inflammation and ARDS in severe COVID-19 [11,15].

No specific research exists on mixed asthma endotypes and COVID-19, highlighting an area for future study [12].

Researchers and clinicians are still striving to understand how COVID-19 interacts with pre-existing conditions like severe asthma. While respiratory viruses are among the most common triggers of asthma exacerbations, their impact on patients varies. The evidence regarding whether asthma increases the risk of SARS-CoV-2 infection or severe disease remains inconclusive and warrants further investigation [16].

## 3. Biologics and Severe Asthma: A Focus on Their Antiviral Activity

Biologic therapies represent a significant advancement in the management of severe asthma. These targeted treatments act on key immune pathways to reduce inflammation and prevent exacerbations. Each biologic has distinct mechanisms of action [17].

### 3.1. Omalizumab

Omalizumab is a humanized recombinant monoclonal antibody approved by the Food and Drug Administration (FDA) and the European Medicines Agency (EMA) as an add-on treatment for patients aged 6 years and older with moderate-to-severe persistent allergic asthma. It is indicated for individuals with an inadequate response to ICS, elevated serum immunoglobulin E (IgE) levels (30 to 1500 IU/mL), and positive specific serum IgE to at least one aeroallergen [18,19].

Omalizumab binds to the Cε3 domain of the fragment crystallizable (Fc) region of circulating IgE, forming inert complexes that do not activate the complement system. This action significantly reduces serum IgE levels. Furthermore, omalizumab inhibits the interaction between IgE and its high-affinity (FcεRI) and low-affinity (FcεRII/CD23) receptors, which are found on the membranes of mast cells, basophils, eosinophils, neutrophils, dendritic cells, and lymphocytes. By doing so, it reduces receptor expression and inhibits the release of inflammatory mediators [20].

In addition, omalizumab acts on IgE bound to B-cell receptors (BCRs). The synthesis of IgE is regulated through the interaction between the IgE-BCR complex and CD21 on B cells. Soluble CD23 (sCD23) induces B cells to produce IgE. Omalizumab disrupts this process by binding to the IgE-BCR complex, thereby preventing IgE synthesis and inducing cellular apoptosis [21].

Omalizumab also enhances the antiviral response in patients with allergic asthma, particularly those with high serum IgE levels who are more susceptible to viral-induced exacerbations. This antiviral effect is attributed to its impact on plasmacytoid dendritic cells (pDCs), which are key producers of interferon (IFN), particularly IFN-α, in response to interactions between virions and Toll-like receptors (TLRs) expressed on their plasma membranes. Compared to healthy individuals, patients with allergic asthma demonstrate an increased expression of FcεRI on dendritic cells and elevated IgE levels. This correlates with a significant down-regulation of TLR expression and a reduced production of IFN-α in response to viral infections. By reducing FcεRI expression and IgE levels, omalizumab helps restore TLR function and enhances the TLR-mediated antiviral response, leading to improved IFN-α production by dendritic cells [22,23].

Finally, following omalizumab treatment, IL-33 levels decrease. This is significant because IL-33 plays a crucial role in the production of inflammatory cytokines, including IL-6, IL-1β, tumor necrosis factor (TNF), and prostaglandin D2 [24].

### 3.2. Dupilumab

Dupilumab is a fully human IgG4 monoclonal antibody that targets the IL-4 receptor alpha (IL-4Rα) subunit, inhibiting the signaling of IL-4 and IL-13, involved in the pathogenesis of several allergic and T2 inflammatory conditions. It is approved by the FDA and the EMA as an add-on maintenance treatment for moderate-to-severe asthma in patients aged 6 years and older with an eosinophilic phenotype or who are oral corticosteroid (OCS)-dependent [25,26,27].

The effects of dupilumab on the immune system may have a crucial impact on viral infections, primarily due to its role in shifting the immune balance from a Th2-dominant profile, associated with impaired antiviral defense and increased susceptibility to infections, to a Th1-dominant profile [28].

A key mechanism by which dupilumab exerts its antiviral effects is the enhancement of IFN-γ production. Increased IFN-γ levels contribute to a more effective immune response against viral infections, potentially aiding in the clearance of persistent viral pathogens [29].

In the context of SARS-CoV-2 infection, IL-13 has been implicated in driving severe COVID-19 outcomes by exacerbating airway inflammation, increasing mucus production, and promoting immune cell recruitment. By inhibiting IL-13 signaling, dupilumab may help mitigate these pathogenic processes, thereby reducing disease severity in COVID-19 patients [30].

Emerging clinical data suggest that dupilumab-treated individuals exhibit a lower incidence and severity of COVID-19 symptoms, further supporting its potential role as an adjunctive therapeutic option in viral respiratory infections. These findings underscore the need for further research into the immunomodulatory effects of dupilumab and its broader implications in antiviral defense, particularly against respiratory viruses such as SARS-CoV-2 [28].

### 3.3. Mepolizumab, Reslizumab, and Benralizumab

Mepolizumab is a humanized IgG1k monoclonal antibody that targets IL-5, preventing its interaction with the IL-5 receptor alpha (IL-5Rα) subunit and thereby suppressing eosinophilic inflammation. It is approved by the FDA and the EMA as an add-on therapy for subjects aged 6 years and older with severe treatment-resistant eosinophilic asthma, characterized by elevated blood eosinophil levels (≥300 cells/µL in the past year) alongside either frequent systemic corticosteroid-requiring exacerbations (≥4 in the past year) or prolonged daily corticosteroid use (equivalent to ≥5 mg/day of prednisolone for six months). Alternatively, eligibility comprises a blood eosinophil count ≥400 cells/µL with at least three steroid-treated exacerbations in the past year [31,32].

Reslizumab is a humanized IL-5 antagonist IgG4κ monoclonal antibody approved by the FDA and EMA as an add-on maintenance treatment for patients aged 18 years and older with severe asthma and an eosinophilic phenotype [33,34].

Benralizumab is an IL-5Rα-directed cytolytic monoclonal antibody (IgG1κ) approved by the FDA as an add-on maintenance treatment for patients aged 6 years and older with severe asthma and an eosinophilic phenotype. The EMA has only approved it for adults (aged ≥ 18 years) [35,36].

IL-5 plays a key role in the regulation and survival of eosinophils, which are involved in immune responses, including antiviral defense [37]. Eosinophils can contribute to the modulation of IFN responses and produce antiviral molecules such as ribonucleases (RNases), including eosinophil-derived neurotoxin (EDN) and eosinophil cationic protein (ECP), which have antiviral properties. These RNases can degrade viral RNA, limiting viral replication and spread. However, excessive eosinophilic inflammation may impair antiviral immunity and exacerbate respiratory viral infections [38]. Mepolizumab, reslizumab, and benralizumab, by targeting IL-5 or its receptor, reduce eosinophil levels and may help regulate immune responses during viral infections. They also act by increasing the ratio of IFN-γ to IL-5 mRNA, which is associated with lower viral shedding and faster disease clearance [39].

### 3.4. Tezepelumab

Tezepelumab is a human IgG2λ monoclonal antibody that targets thymic stromal lymphopoietin (TSLP). It has received approval from both the FDA and the EMA for the add-on maintenance treatment of severe asthma in adults and adolescents aged 12 years and older [40,41].

By inhibiting TSLP, tezepelumab addresses an upstream component in the inflammatory pathway of asthma, offering a novel therapeutic option for patients with severe asthma [42].

Specifically, TSLP is an epithelial “alarmin” that is excessively released in response to viral infections and other triggers. It acts on various immune cells, including PDCs, Th cells, innate lymphoid cells (ILCs), eosinophils, and mast cells, contributing to both T2 and non-T2 inflammatory processes. Its critical role in asthma has been highlighted through clinical studies evaluating the effects of blocking TSLP signaling on inflammation and disease progression [43,44].

Recently, Sverrild et al. investigated the effects of TSLP blockade on host resistance (measured by IFN-β, IFN-λ, and viral load) and airway epithelial inflammatory responses to viral challenge in asthma patients. Bronchoalveolar lavage fluid (BALF) and bronchial epithelial cells were collected from uncontrolled asthma patients before and after 12 weeks of tezepelumab or placebo treatment. Bronchial epithelial cells were then exposed to the viral infection mimic poly(I:C) or infected with Rhinovirus, and inflammatory markers were analyzed. Results showed that tezepelumab treatment reduced IL-33 expression in BALF and bronchial epithelial cells, as well as IL-33 and T2 cytokine (IL-4, IL-13, IL-17A) production in response to poly(I:C). However, TSLP gene expression remained unchanged, and antiviral markers (IFN-β, IFN-λ) and viral load were not affected. In conclusion, tezepelumab may reduce airway epithelial inflammatory responses, particularly IL-33 and T2 cytokines, without impairing antiviral defenses. These findings suggest that inhibiting TSLP may help stabilize bronchial epithelial immune responses to respiratory viruses in asthma [42].

## 4. Safety of Biologics During COVID-19

While the efficacy and safety of monoclonal antibodies have been well established in several clinical trials, their risk profile in patients with active SARS-CoV-2 infection remains poorly understood and requires further investigation.

An overview of studies investigating the use of biologics in asthmatic patients with active SARS-CoV-2 infection is provided in Table 1.

Regarding omalizumab, although none of the studies included in this review provided explicit information on its safety during active SARS-CoV-2 infection, they did not recommend discontinuing biologic therapy in such cases.

Additional insights can be obtained from a prospective study that examined adverse events associated with biologic administration in ten severe asthmatic patients receiving omalizumab. This study reported no adverse events in the enrolled population, further supporting the continued use of omalizumab even in the presence of SARS-CoV-2 infection [71].

Similarly, no significant adverse events have been reported for dupilumab. The studies reviewed highlighted the need for more detailed information on its safety profile during COVID-19. Accordingly, it is reasonable to hypothesize that the lack of this information could be related to poor relevance or the absence of adverse effects [54,61,62]. Only Manti et al. reported side effects in a patient treated with dupilumab during confirmed SARS-CoV-2 infection. Localized redness and swelling at the injection site were the adverse events mentioned, reinforcing the safety of continuing dupilumab treatment during SARS-CoV-2 infection [71].

Regarding mepolizumab, reslizumab, and benralizumab, no notable adverse effects were reported in severe asthmatic patients with COVID-19 across the studies included in our review, supporting the safety of biologics in patients who experienced SARS-CoV-2 infection. Additionally, the literature suggests that patients with allergies and controlled asthma, characterized by eosinophilia, a robust thymic repertoire, and enhanced innate and adaptive immunity, may have partial protection against COVID-19. This immune profile could act as a protective mechanism, supporting the rationale for continuing biologic treatments during SARS-CoV-2 infection [47,48,49,50,51,52,53,54,55,59,63,64,65,66,67,68,69,70,71].

## 5. Limitations

Despite our findings, several limitations must be considered. First, the findings are constrained by methodological heterogeneity, small sample sizes, and a predominance of case reports and series, reducing the statistical power and generalizability. The inclusion of letters, case reports, and observational studies complicates comparative analysis, while the lack of control groups makes it difficult to isolate the effects of biologics from confounding factors such as comorbidities and concurrent therapies. Additionally, most studies lack longitudinal data, limiting insights into long-term outcomes during and after COVID-19 infection.

A critical gap exists in pediatric data, with only a few case reports addressing safety in younger populations.

Furthermore, many studies fail to account for concurrent medications like corticosteroids, which could independently affect COVID-19 outcomes, and offer limited discussion on biologics’ interactions with SARS-CoV-2 pathophysiology—such as ACE2 and TMPRSS2 modulation—weakening the mechanistic rationale.

Future research should focus on well-controlled trials and mechanistic studies to establish causal relationships and assess long-term safety comprehensively.

## 6. Future Directions

The rapid advancement of biologic therapies has significantly transformed the management of severe asthma, offering targeted approaches that address specific inflammatory pathways. However, the efficacy and safety of these therapies cannot solely be determined by clinical trials; experimental research plays a crucial role in uncovering their full biological impact.

Future investigations should focus on several key areas. First, preclinical studies exploring the molecular mechanisms of biologics can provide critical insights into their off-target effects, potential immunogenicity, and long-term interactions with the immune system—aspects that may not be immediately evident in clinical trials. For instance, research into how biologics influence viral immunity, particularly in the context of SARS-CoV-2 infection, remains essential to ensure their safe use during active infections.

Second, real-world evidence studies should be expanded to capture post-marketing data, helping to identify rare adverse events and evaluate the sustained efficacy of biologics over time. These studies can also shed light on how biologics interact with other medications or underlying comorbidities, adding another layer of safety assessment.

Moreover, the development of next-generation biologics, such as small peptides, and metabolite-based biologics, is opening up new avenues for asthma treatment. Understanding their pharmacokinetics, tissue distribution, and cellular effects requires robust experimental models.

Integrating systems biology approaches and artificial intelligence (AI)-driven simulations may further enhance our ability to predict adverse responses and optimize biologic designs.

## 7. Conclusions

The management of severe asthma is a crucial aspect of public health, extending far beyond the immediate challenges posed by SARS-CoV-2 infection. While the COVID-19 pandemic highlighted vulnerabilities in caring for patients with chronic respiratory conditions, it also provided valuable lessons for future preparedness.

Severe asthma requires a comprehensive public health approach to reduce exacerbation, hospitalization, and mortality. Key strategies include improving access to biologics, especially for patients whose conditions are unresponsive to standard therapies, enhancing patient education and self-management through public health initiatives that empower individuals with knowledge about trigger avoidance, proper inhaler use, and the importance of adherence to prescribed treatments and implementing integrated care models, and strengthening surveillance and data collection, with asthma registries playing a vital role in tracking trends, identifying high-risk populations, and guiding policy decisions.

Recognizing the intersection between severe asthma and viral infections, especially in the context of SARS-CoV-2 infection, is essential. Future strategies should include pandemic preparedness plans, with the development of protocols for the continued management of severe asthma during infectious disease outbreaks. These plans should emphasize remote monitoring and telemedicine options, research into viral-triggered exacerbations, effective communication strategies, and vaccine prioritization to ensure individuals with severe asthma receive timely protection against respiratory pathogens.

By focusing on both severe asthma care and future pandemic preparedness, we can build a more resilient healthcare system. Strengthening these dual aspects will not only improve outcomes for asthma patients but also enhance our capacity to respond to emerging infectious diseases.

## Figures and Tables

**Table 1:** Studies investigating the use of biologics in asthmatic patients with active SARS-CoV-2 infection.

Authors, Year of Publication, and Reference	Title	Study Design	Biologic Drug	N. pts, Gender (M, F)	Age (Year)	Aim of the Study	Results/Conclusion	**AEs**
Lommatzsch M et al., 2020 [45]	COVID-19 in a patient with severe asthma treated with Omalizumab	Retrospective observational study	Omalizumab	1 M	52 y	To report a case of COVID-19 during treatment with omalizumab.	Circumstantial evidence suggests that patients with allergic asthma may have a lower risk of developing severe forms of COVID-19. Additionally, omalizumab was shown to enhance antiviral immunity.	NA
Licari A et al., 2020 [46]	Biologic Use in Allergic and Asthmatic Children and Adolescents During the COVID-19 Pandemic	Retrospective observational study	Omalizumab	3, gender not stated	Not stated	To evaluate allergic patients treated with biological therapies during the COVID-19 pandemic.	Continuing treatment with biologics appears to be safe; however, more data are needed to confirm their safety in patients who contract COVID-19.	NA
Eger K et al., 2020 [47]	Poor outcome of SARS-CoV-2 infection in patients with severe asthma on biologic therapy	Prospective observational study	Omalizumab Mepolizumab Reslizumab Benralizumab	9. Omalizumab: 2 (1 M, 1 F). Mepolizumab: 3 (2 M, 1 F). Reslizumab: 1 F. Benralizumab: 2 (1 M, 1 F).	Not stated	To explore incidence of SARS-CoV-2 infection in asthmatic patients treated with biological therapies, the frequencies of asthma exacerbations at COVID-19 diagnosis, the incidence of hospitalization or ventilatory support.	Patients with severe asthma receiving biologics showed a more severe course of COVID-19 compared to the general population. This may be due to comorbidities, the severity of asthmatic airway inflammation, the use of biologics, or a combination of these factors.	NA
Domínguez-Ortega J et al., 2020 [48]	Early experiences of SARS-CoV-2 infection in severe asthmatics receiving biologic therapy	Retrospective observational study	Omalizumab Mepolizumab Reslizumab	7. Omalizumab: 5 F. Mepolizumab: 1 M. Reslizumab: 1 M.	Omalizumab: 49 y, 51 y, 63 y, 32 y, 62 y. Mepolizumab: 50 y. Reslizumab: 50 y	To evaluate the risk and severity of SARS-CoV-2 infection in asthmatic patients treated with biologics.	Biologic treatment was not associated with severe COVID-19.	NA
Haroun-Díaz et al., 2020 [49]	Severe asthma during the COVID-19 pandemic: Clinical observations	Retrospective observational study	Mepolizumab	1 M	55 y	To determine the prevalence and characterization of COVID-19 among patients with severe asthma according to ERS/ATS criteria.	This severe asthmatic patient with COVID-19 under mepolizumab treatment did not develop pneumonia.	NA
Ciprandi G et al., 2020 [50]	Children and adolescents with allergy and/or asthma seem to be protected from coronavirus disease 2019	Retrospective observational study	Mepolizumab	5, 3 M 2 F	Mean age 22.4 y	To present data concerning both COVID-19 in children and adolescents and mepolizumab treatment in patients with severe asthma.	Allergy and controlled asthma could be partially protected from COVID-19. Moreover, corticosteroids and biologics could be reasonably continued.	NA
García-Moguel I et al., 2020 [51]	COVID-19, severe asthma, and biologics	Retrospective observational study	Benralizumab	2, 1 M 1 F	56 y 62 y	To evaluate clinical outcomes in patients treated with benralizumab and SARS-CoV-2 infection	Benralizumab could have a protective role against SARS-CoV-2 infection.	NA
Rial MJ et al., 2020 [52]	Clinical characteristics in 545 patients with severe asthma on biological treatment during the COVID-19 outbreak	Retrospective observational study	Omalizumab Mepolizumab Reslizumab Benralizumab	35. Omalizumab: 14 (4 M,10 F). Mepolizumab: 11 (6 M, 5 F). Reslizumab: 3 (2 M, 1 F). Benralizumab: 7 (2 M, 5 F).	Omalizumab: 46.3 ± 12.2 y. Mepolizumab: 56.4 ± 50 y. Reslizumab: 49 ± 12.1. Benralizumab: 60.2 ± 11.3	To determine the severity of SARS-CoV-2 infection in asthmatic patients treated with biologics.	No association between COVID-19 severity and biologic therapy for asthma was detected.	NA
Heffler E et al., 2020 [53]	COVID-19 in Severe Asthma Network in Italy (SANI) patients: Clinical features, impact of comorbidities and treatments	Retrospective observational study	Omalizumab Mepolizumab Benralizumab	9. Omalizumab: 1 F. Mepolizumab: 7 (2 M, 5 F). Benralizumab: 1 F	Omalizumab: 48 y. Mepolizumab: 45 y, 65 y, 62 y, 66 y, 53 y, 70 y, 51y. Benralizumab: 45 y.	To investigate the incidence of COVID-19 in a population suffering from severe asthma.	Patients treated with mepolizumab showed a higher prevalence of COVID-19 infection compared to patients treated with omalizumab (71% versus 29%).	NA
Adir Y et al., 2021 [54]	COVID-19 risk and outcomes in adult asthmatic patients treated with biologics or systemic corticosteroids: Nationwide real-world evidence	Retrospective observational study	Omalizumab Dupilumab Mepolizumab Reslizumab Benralizumab	50. Omalizumab: 24 (gender not stated). Dupilumab: 3 (gender not stated). Mepolizumab: 13 (gender not stated). Reslizumab: 3 (gender not stated). Benralizumab: 7 (gender not stated).	Not stated	To evaluate the association between biologics, incidence of SARS-CoV-2, and COVID-19 severity.	Biologics were not associated with an increased risk of infection or severe outcomes.	NA
Antonicelli L et al., 2021 [55]	Severe asthma in adults does not significantly affect the outcome of COVID-19 disease: Results from the Italian Severe Asthma Registry	Prospective observational study	Omalizumab Mepolizumab	7. Omalizumab: 3 F. Mepolizumab: 4 (1 M, 3 F).	Omalizumab: 48 y, 65 y, 51 y. Mepolizumab: 40 y, 57 y, 75 y, 56 y.	To evaluate the severity of SARS-CoV-2 infection in asthmatic patients treated with biologics.	No increased risk of SARS-CoV-2 infection or worse outcomes was documented.	NA
Sönmez SC et al., 2021 [56]	COVID-19, Severe Asthma and Omalizumab Therapy: A Case-Based Inquiry into Association, Management, and the Possibility of a Better Outcome	Retrospective observational study	Omalizumab	2, 1 M 1 F	31 y, 36 y.	To present two cases of severe asthma under biologic therapy and to review current research and guidelines on this matter.	Physicians should not follow step-down strategies and continue necessary regimens, including biologics. Good control of asthma could be key in managing COVID-19.	NA
Leru PM et al., 2021 [57]	Real-Life Benefit of Omalizumab in Improving Control of Bronchial Asthma During COVID-19 Pandemic	Retrospective observational study	Omalizumab	1 F	52 y	To describe the case of a female patient, diagnosed with adult-onset asthma, who continued omalizumab during the COVID-19 pandemic.	Treatment with omalizumab should not be stopped during the COVID-19 pandemic.	NA
Paladini E. et al., 2021 [58]	Case Report: Self-Administration of Omalizumab in an Adolescent With Severe Asthma During SARS-CoV-2 Infection	Retrospective observational study	Omalizumab	1 F	16 y	To report the first case in which an adolescent with severe allergic asthma treated with omalizumab switched to self-administration at home while having SARS-CoV-2 infection.	Monoclonal antibody therapy could be safe and should be continued during SARS-CoV-2 infection to prevent asthma exacerbations.	NA
Izquierdo JL et al., 2021 [59]	The impact of COVID-19 on patients with asthma	Retrospective observational study	Omalizumab Mepolizumab Reslizumab Benralizumab	19. Omalizumab: 9 (gender not stated). Mepolizumab: 7 (gender not stated). Reslizumab: 1 (gender not stated). Benralizumab: 2 (gender not stated).	Not stated	To evaluate the impact of COVID-19 in patients with asthma.	Biologic treatment was not associated with increased incidence in admissions and mortality due to SARS-CoV-2 infection.	NA
Aksu K et al., 2021 [60]	COVID-19 in patients with severe asthma using biological agents	Retrospective observational study	Omalizumab Mepolizumab	10. Omalizumab: 9, gender not stated. Mepolizumab: 1 F	Omalizumab: not stated. Mepolizumab: 55 y.	To evaluate the severity of COVID-19 in patients with severe asthma treated with biologics.	Asthmatic patients treated with biologics showed an increased incidence of SARS-CoV-2 infection, but none developed a severe clinical course.	NA
Bhalla A et al., 2021 [61]	Dupilumab, severe asthma airway responses, and SARS-CoV-2 serology	Prospective observational study	Dupilumab	1 F	23 y	To investigate whether dupilumab can contribute to infection or prolonged viral detection by modulating ACE2 or TMPRSS2 expression and anti-SARS-CoV-2 antibody.	Dupilumab used along with corticosteroids for asthma may be safe during the SARS-CoV-2 pandemic. Dupilumab did not affect TMPRSS2 expression, but it may decrease serum IgG and IgM levels.	NA
Tanabe N et al., 2021 [62]	Dupilumab maintenance therapy in an asthmatic patient with coronavirus disease 2019 pneumonia	Retrospective observational study	Dupilumab	1 M	57 y	To present a case who continued dupilumab maintenance therapy before and during COVID-19 and recovered from COVID-19 pneumonia safely without the exacerbation of asthma.	Treatment with dupilumab may be safe and should not be stopped during the COVID-19 pandemic.	NA
Azim A et al., 2021 [63]	Severe acute respiratory syndrome coronavirus 2 infection in those on mepolizumab therapy	Retrospective observational study	Mepolizumab	4, 2 M 2 F	64 y, 61 y, 66 y, 22 y.	To report the outcomes of four patients with COVID-19 while receiving treatment with mepolizumab.	Mepolizuma was not associated with worse clinical outcomes and eosinopenia during SARS-CoV-2 infection.	NA
Aksu K et al., 2021 [64]	COVID-19 in a patient with severe asthma using mepolizumab	Retrospective observational study	Mepolizumab	1 F	55 y	To investigate the COVID-19 course in a patient with severe asthma treated with mepolizumab.	Treatment with mepolizumab may have a protective effect on the course of COVID-19.	NA
Renner A et al., 2021 [65]	COVID-19 in a severe eosinophilic asthmatic receiving benralizumab—a case study	Retrospective observational study	Benralizumab	1 M	41 y	To describe the clinical course of COVID-19 in an asthmatic patient treated with benralizumab.	Benralizumab could protect patients from developing more severe asthmatic exacerbation due to COVID-19.	NA
Matsuno O et al., 2021 [66]	COVID-19 in a Patient With Severe Eosinophilic Asthma on Benralizumab Therapy: A Case Report and Review of Literature	Retrospective observational study	Benralizumab	1 F	60 y	To describe a case of a severe asthmatic eosinopenic patient treated with benralizumab during SARS-CoV-2 infection.	Treatment with benralizumab did not cause a more severe clinical course of SARS-CoV-2 infection.	NA
Ambrosino A., 2021 [67]	Long-Term Follow-Up of a Severe Eosinophilic Asthmatic Patient With Comorbid Nasal Polyposis Hospitalized for SARS-CoV-2 Infection While Receiving Benralizumab: A Case Report	Retrospective observational study	Benralizumab	1 M	59 y	To describe the clinical course of an asthmatic patient treated with benralizumab during SARS-CoV-2 infection.	Biologic therapy contributed to asthma control. Eosinopenia due to benralizumab did not worsen the clinical course.	NA
Papaioannou AI et al., 2022 [68]	SARS-CoV-2 Infection in Severe Asthma Patients Treated With Biologics	Prospective observational study	Omalizumab Mepolizumab Benralizumab	26. Omalizumab: 9 (7 M, 2 F). Mepolizumab: 16 (4 M, 12 F). Benralizumab: 1 F.	Omalizumab: mean age 55 y. Dupilumab: mean age 57.5 y Benralizumab: 52 y.	To evaluate risks of SARS-CoV-2 infection and COVID-19 severity in sever asthmatic patients.	Biologic treatments were not associated with an increased risk of SARS-CoV-2 infection. Apparently, the infection was associated with a greater risk of hospitalization, particularly in patients treated with mepolizumab.	NA
Votto M et al., 2022 [3]	Safety of biological therapy in children and adolescents with severe asthma during the COVID-19 pandemic: a case series	Retrospective observational study	Omalizumab Mepolizumab	4. Omalizumab: 2 F. Mepolizumab: 2 (1 M, 1 F).	Omalizumab: mean age 15 y. Mepolizumab: mean age 23.7 y.	To evaluate clinical courses in children and adolescents with severe asthma treated with biological therapies during the COVID-19 pandemic.	Well-controlled asthma does not increase the risk of severe COVID-19. Biological therapies could have a protective role in acquiring SARS-CoV-2 and developing severe COVID-19.	NA
Kroes JA et al., 2022 [69]	Administration of benralizumab in a patient with severe asthma admitted to the intensive care unit with COVID-19 pneumonia: case report	Retrospective observational study	Benralizumab	1 F	64 y	To describe the clinical course of an asthmatic patient treated with benralizumab during SARS-CoV-2 infection.	Benralizumab was associated with a self-limiting eosinopenia. Its administration is considered a safe option for patients with severe asthma during COVID-19 infection.	NA
Francis CHR et al., 2022 [70]	COVID-19 in the absence of eosinophils: The outcome of confirmed SARS-CoV-2 infection whilst on treatment with benralizumab	Retrospective observational study	Benralizumab	24, 10 M 14 F	46.5 ± 12.6 y	To report the outcomes of 24 patients with severe asthma who had confirmed SARS-CoV-2 infection whilst under treatment with benralizumab.	The eosinophil count did not impact the severity of SARS-CoV-2 infection.	NA
Manti S et al., 2023 [71]	Safety of biologics in severe asthmatic patients with SARS-CoV-2 infection: A prospective study	Prospective observational study	Omalizumab Dupilumab Mepolizumab	21. Omalizumab: 10 (gender not stated). Dupilumab: 9 (gender not stated). Mepolizumab: 2 (gender not stated).	Not stated	To describe the clinical course of severe asthmatic patients treated with biological therapies.	Biological therapies during COVID-19 are safe and not associated with more severe infection.	Redness and swelling were observed at the injection site in a patient treated with dupilumab.

(ACE2, angiotensin-converting enzyme 2; AEs, adverse events; ATS, American Thoracic Society; COVID-19, coronavirus disease 2019; ERS, European Respiratory Society; F, female; IgG, immunoglobulin G; IgM, immunoglobulin M; M, male; N, number; NA, not available; Pts, patients; SARS-CoV-2, severe acute respiratory syndrome coronavirus 2; TMPRSS2, transmembrane protease serine 2).

## Data Availability

No new data were created or analyzed in this study.
